# Clinical Efficacy of Extended Transforaminal Endoscopic Lumbar Foraminotomy Compared with the Conventional Technique

**DOI:** 10.3390/jcm14186446

**Published:** 2025-09-12

**Authors:** Yong Ahn, Han-Byeol Park, Seong Son, Byung-Rhae Yoo

**Affiliations:** 1 Department of Neurosurgery, Kyung Hee University Hospital at Gangdong, Kyung Hee University College of Medicine, Seoul 05278, Republic of Korea; 2 Department of Neurosurgery, Gachon University Gil Medical Center, Incheon 21565, Republic of Korea; phbsgood@gilhospital.com (H.-B.P.); sonseong@gilhospital.com (S.S.); byungryoo@gilhospital.com (B.-R.Y.)

**Keywords:** endoscopic surgical procedures, foraminal stenosis, lumbar vertebrae, transforaminal, foraminotomy

## Abstract

**Objectives**: Transforaminal endoscopic lumbar foraminotomy (TELF) is an emerging minimally invasive surgical technique for lumbar foraminal stenosis. However, its effectiveness is debated because of concerns regarding adequate decompression and its long-term consistency. This study introduced the extended form of TELF, an advanced technique, to provide more extensive decompression using the same approach. Thus, this study aimed to describe the surgical technique and clinical outcomes of this technique. **Methods**: This retrospective cohort study included patients who underwent conventional (*n* = 67) or extended (*n* = 64) TELF. The surgical procedure involved a transforaminal approach with endoscopic decompression, including the removal of the tip of the superior articular process, foraminal ligament, and ligamentum flavum (conventional group), or additional decompression, involving the isthmus and portions of the superior and inferior pedicle walls (extended group). Clinical outcomes were assessed using the visual analog pain scale, Oswestry disability index, and modified Macnab criteria. **Results**: Despite the longer surgical duration, the extended TELF group tended to show better outcomes in terms of the VAS and ODI scores at the early and final 2-year follow-ups (*p* < 0.05). The overall success rates were 92.19% and 85.07% in the extended and conventional groups, respectively. No difference was observed in surgical complications between the two groups. **Conclusions**: Extended TELF, a refined endoscopic technique, achieves better effects than conventional TELF with a lower risk of nerve root irritation by creating a sufficiently safe resection margin. The results support the use of an extended TELF as an advanced form of endoscopic foraminal decompression.

## 1. Introduction

Lumbar foraminal stenosis (LFS) is often considered a candidate for surgical procedures owing to its potential to cause chronic intractable radicular pain despite extensive conservative treatment. Exiting nerve root (ENR) compression at the foraminal or extraforaminal zone from hypertrophic superior articular process (SAP), thickened ligamentum flavum (LF), herniated disk, collapsed disk height, shoulder osteophytes can cause unbearable radiculopathy [1,2]. The current standard surgical approach is open foraminal decompression surgery, with or without instrumented fusion. However, considerable postoperative morbidities make open surgery for LFS challenging. Moreover, extensive open surgery may cause decompression-related complications [3,4,5] resulting from the irritation of the dorsal root ganglion or incomplete decompression and fusion-related problems [6,7,8,9], such as unnecessary loss of motion and adjacent segment disease.

Endoscopic spine surgery has transitioned into a representative form of minimally invasive spine surgery and has demonstrated its efficacy in numerous randomized controlled trials [10,11,12,13,14,15,16] and meta-analyses [17,18,19,20,21,22,23,24,25]. Among these surgical techniques, TELF has been recognized as an effective endoscopic technique for LFS treatment. Despite its advantages, concerns regarding its ability to achieve decompression equivalent to open surgery persist. Additionally, the rebound phenomenon is caused by degenerative changes, including disk space narrowing, redundant disks, and ligament hypertrophy, which occur over time [26]. These limitations have inhibited the wide adoption of TELF in endoscopic lumbar procedures. To overcome these challenges, an advanced version of TELF, termed extended TELF, was developed in this study. This technique provides a more comprehensive decompression by targeting a wider range of anatomical structures. Extended TELF intends to provide an additional safe resection margin for full-scale nerve root decompression for immediate complete decompression and long-term effectiveness in preventing restenosis.

This study aimed to describe the surgical technique for extended TELF and compare its clinical effectiveness with that of conventional TELF.

## 2. Materials and Methods

### 2.1. Study Design and Patient Selection

This retrospective cohort study included patients who underwent extended (*n* = 64) or conventional (*n* = 67) TELF between January 2021 and December 2022. The choice of surgical technique was determined according to the surgeon’s preference: A.Y. performed extended TELF, whereas S.S. and Y.B.R. preferred conventional TELF.

The following inclusion criteria were adapted from the conventional TELF study: (1) patients diagnosed with LFS presenting with radiculopathy; (2) symptoms refractory to conservative management for at least 6 weeks, including physical therapy, medication, and selective nerve root block; (3) radiological confirmation of foraminal stenosis by magnetic resonance imaging and/or computed tomography, defined as moderate-to-severe foraminal narrowing (grade 2 or 3) with nerve root compression based on the foraminal stenosis grading system [27]; and (4) absence of segmental instability as confirmed by dynamic X-ray imaging.

The exclusion criteria were as follows: intracanalicular central stenosis or lateral recess stenosis without foraminal involvement; spinal instability requiring fusion; coexisting pathologies such as tumors, infections, or acute inflammatory conditions; and history of surgical intervention at the same level, resulting in significant scarring that precluded endoscopic access.

### 2.2. Surgical Techniques

The surgical procedures were performed through a percutaneous transforaminal endoscopic approach under local anesthesia. Patients were positioned prone on a radiolucent table, with conscious sedation induced as necessary. The skin entry point was identified based on preoperative imaging and was typically located 5–10 cm lateral to the midline, allowing an optimal trajectory to the foraminal space. Under fluoroscopic guidance, a spinal needle was then introduced to target the SAP, followed by sequential dilation and placement of a working cannula.

**Figure 1:**
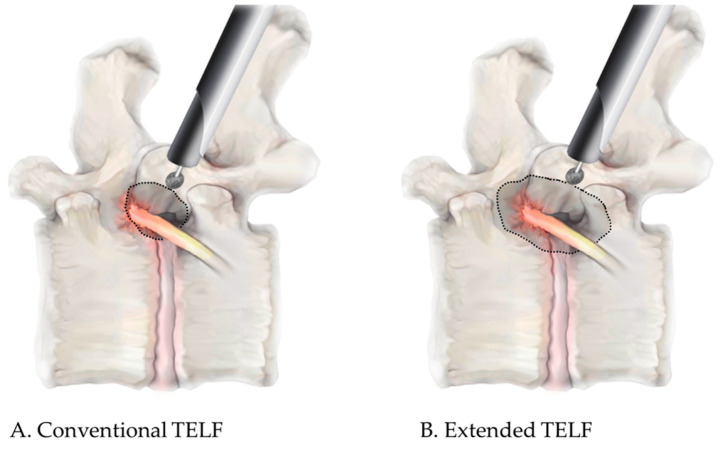
Schematic comparison of the conventional and extended TELF in the lateral view. (**A**). Conventional TELF involves the removal of the tip of the superior articular process and the ligamentum flavum to decompress the exiting nerve root from the axillary zone to the lateral exit zone. (**B**). Extended TELF further expands the decompression range by resecting up to the isthmus and the upper pedicle wall superiorly and down to the part of the inferior pedicle and transverse process inferiorly, providing a sufficient resection margin around the exiting nerve root.

**Figure 2:**
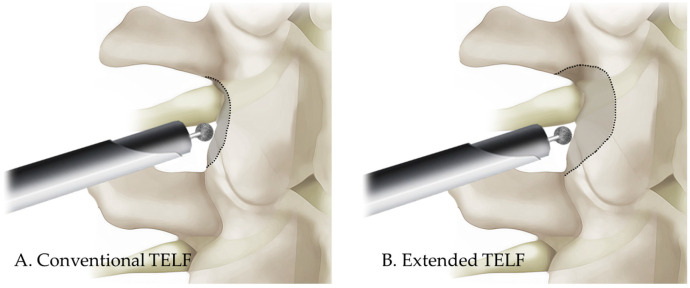
Schematic comparison of the conventional and extended TELF in the posterior view. (**A**). Conventional TELF mainly focuses on full-scale decompression throughout the trajectory of the exiting nerve root from the proximal axillary zone to the lateral exit zone. (**B**). Extended TELF covers a wider area, including the vertical and medial safety resection margins, including the superior and inferior pedicle wall and the medial aspect of the facet joint.

In conventional TELF, foraminal decompression is achieved using a stepwise approach. The initial stage involved foraminal unroofing, wherein a high-speed burr was used to undercut the SAP, which exposes the foraminal ligament and LF. The foraminal ligament was carefully removed using endoscopic punches and forceps to release the ENR. Additional decompression included resection of the hypertrophic LF and osteophytes adjacent to the neural structures. Hemostasis was achieved using a bipolar radiofrequency probe, and free mobilization of the nerve root indicated sufficient neural decompression (Figure 1A and Figure 2A). Upon completion of the procedure, the working cannula was withdrawn, and a simple dressing was applied to the small incision site.

**Figure 3:**
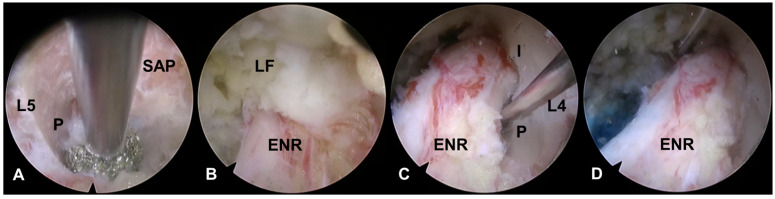
Endoscopic images of extended transforaminal endoscopic lumbar foraminotomy. (**A**). Initial endoscopic view during resection of the lower pedicle wall (P) and hypertrophic superior articular process (SAP) using an endoscopic burr (L4–5, right side). (**B**). Status after bony foraminal unroofing. Note the residual ligamentum flavum (LF) and the exposed exiting nerve root (ENR). (**C**). Extended decompression of the isthmus (I) and upper pedicle wall (P), contributing to a broader foraminal decompression. (**D**). Final endoscopic view demonstrating full-scale foraminal decompression with an adequate resection margin.

The extended TELF incorporated additional modifications to enhance the foraminal decompression. Compared with the conventional technique, extended TELF used a steeper approach angle targeting the extraforaminal zone of the disk space to improve surgical access and visualization. Bone resection was extended beyond the SAP tip to include portions of the isthmus and pedicle walls, allowing for more comprehensive expansion of the foramen (Figure 3). This approach facilitates greater decompression, particularly in cases of severe foraminal stenosis, where conventional SAP resection is insufficient. Additional soft tissue decompression was performed to address the adhesions and inflammatory changes around the nerve root. The nerve was carefully mobilized within the axillary epidural space, ensuring complete release from the surrounding structures. After the proximal decompression of the ENR, the adequacy of the distal decompression was assessed. If compression due to a shoulder osteophyte or transverse process (or sacral alar in case of S1 vertebra) is identified, these structures should be meticulously resected to decompress the lateral exit zone. This additional decompression ensures total decompression of the ENR, optimizes surgical outcomes, and minimizes the residual nerve root compression. The endpoint of the procedure was established by confirming the free movement and pulsation of the ENR under endoscopic visualization (Figure 1B and Figure 2B).

Both conventional and extended TELF were performed under continuous endoscopic guidance, which minimizes disruption to the surrounding anatomical structures while achieving effective decompression. Postoperative assessments included immediate imaging to evaluate the efficacy of decompression and monitoring for transient dysesthesia, which was more often observed following extended TELF because of the greater extent of neural mobilization.

### 2.3. Clinical Outcome Measures

Clinical outcomes were assessed preoperatively and at multiple postoperative time points: 6 weeks, 6 months, 1 year, and 2 years. Pain intensity was evaluated using a visual analog scale (VAS) for leg and back pain, with patients reporting their perceived pain levels on a 10-point scale at each follow-up visit. Functional status was assessed using the Oswestry disability index (ODI) [28], which quantifies the degree of disability related to daily activities. This allows for an objective assessment of improvements in patient mobility and functional recovery following surgery.

Patient-reported satisfaction and symptom relief were categorized according to the modified Macnab criteria [29], which classify outcomes as excellent, good, fair, or poor based on the extent of symptom resolution and the patient’s ability to resume normal activities. Postoperative flare-ups, defined as a transient worsening of pain or the onset of new dysesthesia occurring within the first 4 weeks after surgery, were also monitored. These flare-ups were documented through patient-reported symptoms and clinical evaluations, with additional interventions provided as needed to manage discomfort and optimize recovery.

### 2.4. Statistical Analysis

Preoperative and postoperative outcomes were compared using paired *t*-tests or repeated-measures analysis of variance with Bonferroni correction for multiple comparisons. Significance was set at *p* of <0.05.

## 3. Results

### 3.1. Demographic and Operative Data

The extended TELF group (*n* = 64) included 24 male and 40 female patients, with a mean age of 69.11 (range, 45–86) years. The conventional TELF group (*n* = 67) included 30 male and 37 female patients, with a mean age of 69.60 (range, 43–88) years. No significant differences were found in sex, age, weight, height, and body mass index between the groups. In both groups, the most frequently operated level was L5–S1, accounting for 46.56% of cases, followed by L4–5 at 40.46%. The mean operative times of the extended and conventional TELF groups were 60.27 ± 15.81 (range, 30–100) and 54.57 ± 13.71 (range, 33–90) min, respectively. The mean operative time was significantly longer in the extended TELF group than in the conventional TELF group (*p* < 0.05), reflecting the additional bone resection and neural mobilization required for wider foraminal decompression. Despite the longer surgical duration, no significant differences in hospital stay were found between the two groups, as most patients were discharged within 24 h after surgery. The demographics and operative data are presented in Table 1.

### 3.2. Clinical Outcomes

Overall, the mean (± standard deviation) VAS score for radicular leg pain improved from 8.09 ± 0.75 to 1.98 ± 1.18 and from 8.09 ± 0.68 to 2.39 ± 1.11 in the extended and conventional TELF groups, respectively. The mean ODI improved from 69.90% ± 10.78% to 19.14% ± 16.58% and from 70.07% ± 10.62% to 23.95% ± 10.46% in the extended and conventional TELF groups, respectively.

Both groups demonstrated significant improvements in pain scores and functional status. The VAS score decreased by 6.19 points in the extended TELF group and 6.15 in the conventional TELF group. Similarly, the ODI improved by 50.76% in the extended TELF group compared with 46.13% in the conventional TELF group. Compared with the conventional group, the extended TELF group showed significantly better outcomes based on VAS and ODI scores at 6 weeks postoperatively (*p* < 0.05). Between 6 months and 1 year, both groups demonstrated continuous improvement, with no significant differences. At the final 2-year follow-up, the extended TELF group again exhibited a significantly better outcome based on VAS scores than the conventional group (*p* < 0.05), indicating a favorable long-term trend (Table 2).

**Figure 4:**
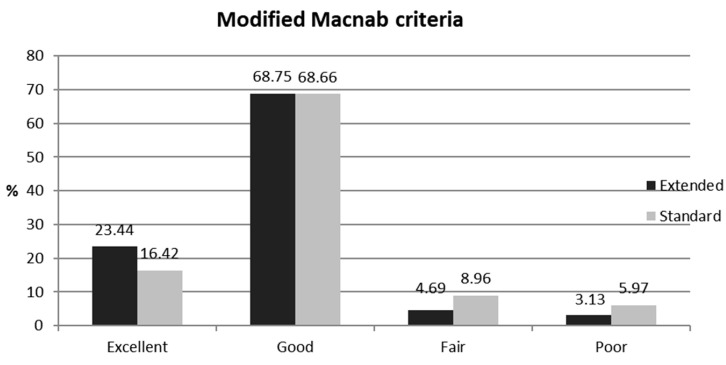
Global results according to the modified Macnab criteria. An excellent or good outcome was observed in 59 of the 64 patients (92.19%) in the extended TELF group and in 57 of the 67 patients (85.07%) in the conventional TELF group. However, no statistical differences were found between the groups.

At the final follow-up, the overall success rates (excellent or good outcomes) were 92.19% (extended group) and 85.07% (conventional group). Although the extended TELF group generally showed a higher proportion of favorable outcomes, the difference between the two groups was not significant (Figure 4).

### 3.3. Complications and Reoperations

Intraoperative dural tears requiring repair occurred in two cases in the conventional TELF group, whereas no dural tears or infections were observed in the extended TELF group. Dural tears were successfully repaired under endoscopic visualization using TachoSil^®^ (Corza Medical, Zurich, Switzerland) and fibrin sealant. Postoperative dysesthesia was reported in 3 of 64 (4.69%) patients in the extended TELF group and in 5 of 67 (7.46%) patients in the conventional TELF group, and symptoms generally resolved with conservative treatment. During the follow-up period, one patient in the extended TELF group required conversion to open decompression and fusion surgery because of recurrent radicular symptoms. Conversely, two patients in the conventional TELF group underwent additional open surgery. Throughout the follow-up period, all patients underwent periodic dynamic radiography, and no newly developed instability or hypermobility was identified in either study group (Table 2).

## 4. Discussion

### 4.1. Clinical Outcome Data

The clinical outcomes of both surgical approaches demonstrated a significant reduction in pain and functional disability, as reflected in the improvements in the VAS and ODI scores. A reduction in pain scores by >50% [30] and an improvement in ODI scores by at least 30% [31,32] are generally considered indicators of clinically relevant effectiveness. These findings align with the results of previous studies on transforaminal endoscopic decompression techniques, supporting the role of endoscopic lumbar foraminotomy in providing effective and sustained symptom relief [33,34]. The final modified Macnab score and complication rate were not significantly different between the two groups, indicating that both techniques are comparably effective in the treatment of LFS. The extended TELF group demonstrated relatively superior outcomes in VAS and ODI scores at the 6-week follow-up and significantly better VAS scores at the 2-year follow-up, indicating the clinical superiority of the extended technique. The extent of decompression achieved may have contributed to the superior outcomes observed in the extended TELF group during the early postoperative period and at the 2-year follow-up. The sufficient resection margin in the extended approach may have minimized postoperative flare or rebound pain caused by initial nerve swelling and inflammatory responses, thereby contributing to better early clinical results. In the long term, this broader decompression helped prevent restenosis associated with progressive degenerative changes, such as disk degeneration and space narrowing, which often develop over time. These factors may explain the relatively favorable outcomes observed at the final follow-up in the extended group. Taken together, extended TELF offers more stable and effective results than the conventional technique in the early as well as long-term postoperative periods. Despite the relatively longer operative time, extended TELF provides more complete decompression and superior clinical outcomes.

### 4.2. Conventional TELF and Its Problems

Conventional TELF was originally developed to achieve full-scale decompression of the ENR, typically extending from the axillary portion of the foramen to the lateral exit zone. Although effective in many cases, it often leaves residual compression at the upper or lower margins of the foramen, particularly in patients with more complex or vertically extended foraminal stenosis. Therefore, despite its favorable clinical outcomes, this full-endoscopic foraminal decompression technique remains challenging, even for experienced endoscopic spine surgeons. A primary concern is the relatively high incidence of postoperative dysesthesia or flare, which is often reported in patients undergoing this procedure [33,35]. Additionally, conventional TELF has shown limited effectiveness in addressing vertical stenosis caused by disk space narrowing because it primarily focuses on foraminal decompression rather than disk height restoration. Another significant drawback is pain recurrence within 1–2 years postoperatively, commonly referred to as the rebound phenomenon. This delayed symptom recurrence has raised concerns regarding the long-term durability of conventional TELF. Given these limitations, endoscopic foraminotomy has been adopted at a slower pace than endoscopic laminectomy or discectomy within the spinal surgical community. Its relatively gradual acceptance and slow development have highlighted the need for a more refined and practical endoscopic technique that can effectively address these challenges.

### 4.3. Extended TELF: A Solution with Strengths and Challenges

Conversely, extended TELF provides a more comprehensive decompression corridor from the upper pedicle with the shoulder of the ENR through the isthmus and axillary zone, across the lateral exit zone, and down to the lower pedicle. This vertical extension allows for the full release of the ENR from all surrounding bony and soft tissue constraints, which effectively addresses pathologies that may not be completely resolved by conventional TELF. This advanced and wider decompression technique offers several potential advantages that may help in improving clinical outcomes and patient satisfaction.

First, extended TELF allows for a more extensive decompression, effectively preventing incomplete decompression and hidden remaining pathologies. Thoroughly addressing foraminal stenosis ensures immediate pain relief and durability, thereby reducing the likelihood of residual symptoms. It can also minimize transient postoperative irritation and neural edema, which are common causes of postoperative flares or rebound pain. Mitigating these factors improves early postoperative recovery and reduces patient discomfort in the immediate postoperative period.

Second, this technique is crucial in addressing a broader spectrum of foraminal pathologies, such as vertical and dynamic foraminal stenosis. Although conventional TELF has demonstrated clinical effectiveness for typical foraminal stenosis, its efficacy in vertically extended stenosis remains limited. Accordingly, Sugiura et al. [36] reported that conventional TELF showed limited effectiveness in treating vertical foraminal stenosis owing to its constrained decompression corridor. Conversely, extended TELF enables vertical decompression from the upper pedicle to the lower pedicle, thereby providing sufficient clearance along the full height of the foramen. This anatomical reach allows for effective neural decompression even in vertically oriented stenosis. Moreover, extended TELF is advantageous for the treatment of dynamic foraminal stenosis. Securing an adequate foraminal space through circumferential decompression reduces positional nerve compression, which often worsens with axial loading or postural changes. Consequently, patients benefit from more stable symptom relief, quicker return to daily living activities, and improved long-term functional outcomes.

Finally, extended TELF may contribute to the long-term prevention of foraminal restenosis. Progressive degenerative changes and gradual disk space narrowing can lead to recurrent foraminal stenosis, necessitating additional interventions. Ensuring sufficient foraminal dimensions may help sustain surgical benefits and reduce the risk of reoperations.

The findings of this study validate these theoretical advantages and demonstrate the clinical efficacy of extended TELF in achieving thorough decompression, alleviating postoperative discomfort, and preventing early and late complications associated with foraminal stenosis. Given these advantages, extended TELF presents strongly as a new standard for endoscopic foraminal decompression procedures.

Despite its clinical benefits, extended TELF has some limitations. It is inherently more technically demanding than open or microscopic decompression and requires higher levels of endoscopic skills and anatomical understanding. In addition, the operative time is generally extended owing to the expanded decompression area and the need for meticulous bleeding control. These drawbacks highlight the importance of adequate training and experience in adopting extended TELF. However, the novelty of extended TELF lies in achieving this full-scale decompression via a minimally invasive endoscopic transforaminal route, thereby minimizing muscle dissection, mitigating blood loss, and reducing postoperative morbidity.

### 4.4. Technical Pearls for Success

The successful execution of extended TELF relies on several critical technical elements. First, taking an appropriate transforaminal trajectory into the pathological foramen is a key determinant of surgical success. Thus, the recommendation is to begin with a steep approach angle, typically ≥50°, and gradually shift toward a more horizontal orientation as the decompression proceeds. This dynamic adjustment of the working angle improved the efficiency of accessing and decompressing the foraminal space. Conversely, initiating the procedure with a shallow angle may hinder adequate exposure and limit the effectiveness of foraminal decompression. This approach differs from the trajectory commonly employed in transforaminal endoscopic discectomy for intracanal lumbar disk herniation, in which a more horizontal path is often preferred. Furthermore, positioning the final working sheath just outside the foramen, consistent with the outside-in technique, allows for the safe and controlled initiation of foraminal decompression.

Second, maintaining precise anatomical orientation throughout the procedure is critical for the success of endoscopic decompression. Unlike open surgery, endoscopic procedures provide only a limited and segmented visual field, making it difficult to perceive the full anatomical context. Therefore, key anatomical landmarks must be identified as reference points to avoid disorientation during the procedure. The most reliable landmarks are typically bony structures, including the disk surface, lower pedicle, SAP, facet joint synovium, isthmus, and the upper pedicle. Sequentially confirming these landmarks and proceeding with decompression using instruments such as endoscopic burrs and punches help surgeons achieve clean and effective decompression without injuring the ENR. In contrast, relying too heavily on soft tissue orientation during decompression may increase the risk of disorientation and potential neural injury and should therefore be approached with caution.

Third, a sufficient bony resection margin must be achieved to obtain satisfactory clinical outcomes. Beyond simple decompression and nerve release, an ample decompressive space is crucial in preventing postoperative issues, such as incomplete decompression due to adhesion or restenosis resulting from progressive degenerative changes. Meticulously removing all the surrounding soft tissue around the exposed nerve root and leaving only clean bony margins can substantially reduce the risk of postoperative fibrotic adhesion. Adhesions are more likely to form between the neural tissues and residual soft tissues, such as ligaments or fibrotic bands. Conversely, the likelihood of adhesion between the neural tissue and bone was significantly lower. This concept is supported by previous studies demonstrating reduced perineural fibrosis and improved clinical outcomes when bare bone surfaces are preserved adjacent to neural structures during spinal decompression procedures [37]. This strategy ensures both the immediate and long-term efficacy of the extended TELF technique.

Finally, a clear endoscopic visual field must be maintained throughout the procedure to ensure surgical safety and efficiency. Because endoscopic spine surgery relies on water-based two-dimensional imaging, even minor intraoperative bleeding or instrument shadowing can obstruct the view and increase the risk of neural injury. In particular, slight epidural or bone bleeding can lead to the so-called red sun phenomenon, where the endoscopic field turns red, making safe manipulation nearly impossible. To prevent this, the irrigation pressure must be regulated using a controlled pressure pump, irrigation solutions mixed with epinephrine or tranexamic acid utilized, and meticulous hemostasis performed with a tip-controllable radiofrequency probe. If bleeding persists despite these measures, hemostatic agents should be applied using a soaking technique for approximately 2 min before resuming the procedure. These strategies are vital for ensuring a safe and smooth surgical process under endoscopic visualization.

### 4.5. Future Perspectives

Despite the promising outcomes of extended TELF, specific technical barriers must be addressed to further optimize the procedure. Three primary challenges—the surgical approach, optics, and surgical instruments—remain critical areas for future improvement. Among these, advancements in surgical instruments are the most urgent. The development of steerable and articulation of endoscopic instruments would significantly enhance the surgeon’s ability to navigate within the confined transforaminal space, allowing for more effective decompression with a reduced risk of neural injury [38]. In addition, innovations in optical systems, including high-resolution endoscopic imaging and real-time three-dimensional visualization, may further improve the intraoperative precision. As technology evolves, the integration of robotic assistance and navigation systems driven by artificial intelligence holds promise for overcoming the current limitations and reducing the learning curve associated with endoscopic spine surgery.

### 4.6. Study Limitations

Although this study provides valuable insights into the clinical benefits of extended TELF, several limitations remain. First, this study followed a retrospective cohort design rather than a randomized controlled trial, which inherently increases the risk of bias, indicating the most significant limitation. Second, two surgeons performed conventional TELF procedures, whereas a single surgeon performed all extended TELF procedures. This design may have introduced a confounding effect and could contribute to bias. Nevertheless, in this study, each surgeon possessed a sufficient skill level and performed the procedures under strictly standardized indications, thereby minimizing the potential confounding effect. Third, the single-center setting limits the generalizability of the findings; thus, a multicenter approach involving diverse patient populations and multiple surgeons is necessary to establish reproducibility [33,34]. Fourth, the relatively small sample size limits the statistical power of the conclusions, prompting the need for further studies with larger cohorts and extended follow-up periods to assess long-term outcomes. To further minimize bias and improve the reliability of future research, well-designed, multicenter, randomized controlled trials involving multiple surgeons are warranted.

## 5. Conclusions

Extended transforaminal endoscopic foraminotomy represents an advanced minimally invasive technique for the treatment of LFS. Although it requires a longer operative time than the conventional approach, the extended technique demonstrated greater improvements in pain relief at the early (6-week) follow-up and maintained significantly better VAS scores at the 2-year follow-up. These findings indicate a potential clinical advantage of extended endoscopic decompression; however, to validate its efficacy and eliminate confounding, well-designed multicenter randomized controlled trials are warranted.

## Figures and Tables

**Table 1:** Patient demographics and perioperative data.

	Extended TELF	Conventional TELF	Total	*p*-Value
No. of patients	64	67	131	
Sex ratio (M:F)	24:40	30:37	54:77	NS
Mean age (years)	69.11 ± 7.91	69.60 ± 10.21	69.36 ± 9.13	NS
Mean BMI (kg/m^2^)	23.54 ± 3.46	24.02 ± 3.57	23.77 ± 3.51	NS
Operative level				NS
L1–L2	0	0	0	
L2–L3	2	1	3	
L3–L4	7	7	14	
L4–L5	26	27	53	
L5–S1	29	32	61	
Operative time (min)	60.27 ± 15.81	54.57 ± 13.71	57.35 ± 14.99	* 0.0291

TELF = Transforaminal endoscopic lumbar foraminotomy; BMI = Body mass index; NS = Not significant; * = Statistically significant.

**Table 2:** Comparison of clinical outcomes.

	Extended TELF	Conventional TELF	*p*-Value
VAS			
Preop	8.09 ± 0.75	8.19 ± 0.68	NS
Postop 6 weeks	3.14 ± 1.60	3.73 ± 1.68	* 0.0419
Postop 6 months	2.80 ± 1.89	3.30 ± 1.91	NS
Postop 1 year	1.94 ± 1.28	2.16 ± 1.34	NS
Postop 2 years	1.98 ± 1.18	2.39 ± 1.11	* 0.0456
ODI (%)			
Preop	69.90 ± 10.78	70.07 ± 10.62	NS
Postop 6 weeks	27.83 ± 17.47	33.95 ± 16.96	* 0.0435
Postop 6 months	27.16 ± 17.29	28.69 ±16.74	NS
Postop 1 year	19.12 ± 16.17	21.35 ± 16.31	NS
Postop 2 years	19.14 ± 16.58	23.94 ± 15.46	0.0887
Modified Macnab (%)			
Excellent	23.44	16.42	NS
Good	68.75	68.66	NS
Fair	4.69	8.96	NS
Poor	3.13	5.97	NS
Dysesthesia	3 (4.69%)	5 (7.46%)	NS
Dural tear	0 (0%)	2 (2.99%)	NS
Revision surgery	1 (1.56%)	2 (2.99%)	NS

TELF = Transforaminal endoscopic lumbar foraminotomy; VAS = Visual analog pain scale; ODI = Oswestry Disability Index; NS = Not significant; * = Statistically significant.

## Data Availability

The data presented in this study are available upon request from the corresponding author.

## Abbreviations

The following abbreviations are used in this manuscript:

ENR	Exiting nerve root
LF	Ligamentum flavum
LFS	Lumbar foraminal stenosis
ODI	Oswestry disability index
TELF	Transforaminal endoscopic lumbar foraminotomy
VAS	Visual analog scale
